# Self-harm in young people with perinatal HIV and HIV negative young people in England: cross sectional analysis

**DOI:** 10.1186/s12889-019-7424-7

**Published:** 2019-08-27

**Authors:** Julie Copelyn, Lindsay C. Thompson, Marthe Le Prevost, Hannah Castro, Kate Sturgeon, Katie Rowson, Susie Brice, Caroline Foster, Diana M. Gibb, Ali Judd, on behalf of the Adolescents and Adults Living with Perinatal HIV (AALPHI) Steering Committee, S. Brice, A. Judd, M. Le Prevost, A. Mudd, F. Parrott, K. Rowson, K. Sturgeon, M. Conway, K. Doerholt, D. Dunn, C. Foster, D. M. Gibb, S. Kinloch, N. Klein, H. Lyall, D. Melvin, K. Prime, C. Sabin, M. Sharland, C. Thorne, P. Tookey, C. Diaz Montana, K. Fairbrother, M. Rauchenberger, N. Tappenden, S. Townsend, D. M. Gibb, D. Mercey, F. Boag, P. Seery, M. Clapson, V. Noveli, A. Callaghan, E. Menson, A. Walley, E. Cheserem, E. Hamlyn, R. Gilson, T. Peake, S. Liebeschuetz, J. Daniels, T. Fernandez, S. Kinlock de Loes, S. Storey, S. Paulus, A. Riordan, J. Daglish, C. Robertson, J. Bernatonlene, L. Hutchinson, M. Gompel, L. Jennings, M. Dowie, S. O’Riordan, W. Ausalut, S. Bandi, P. McMaster, C. Murphy, M. Chaponda, S. Paulus, C. Dufton, B. Oliver, J. Marsh, I. Clowes, M. Kiwanuka, B. Chipalo, Ali Judd

**Affiliations:** 1 Medical Research Council (MRC) Clinical Trials Unit at University College London (UCL), 90 High Holborn, WC1V 6LJ, London, UK; 2 Imperial College Healthcare NHS Trust, London, UK

**Keywords:** Self-harm, Perinatal, HIV, Young people, Adolescents, England, Self-esteem

## Abstract

**Background:**

Self-harm in adolescents is of growing concern internationally but limited evidence exists on the prevalence of self-harm in those living with HIV, who may be at higher risk of poor mental health outcomes. Therefore our aim was to determine the prevalence and predictors of self-harm among young people with perinatally-acquired HIV (PHIV) and HIV negative (with sibling or mother living with HIV) young people living in England.

**Methods:**

303 PHIV and 100 HIV negative young people (aged 12–23 years) participating in the Adolescents and Adults Living with Perinatal HIV cohort study completed an anonymous self-harm questionnaire, as well as a number of standardised mental-health assessments. Logistic regression investigated predictors of self-harm.

**Results:**

The median age was 16.7 years in both groups, and 40.9% of the PHIV and 31.0% of the HIV negative groups were male. In total 13.9% (56/403) reported having ever self-harmed, with no difference by HIV status (*p* = 0.089). Multivariable predictors of self-harm were female sex (adjusted odds ratio (AOR) 5.3, (95% confidence interval 1.9, 14.1), *p* = 0.001), lower self-esteem (AOR 0.9 (0.8, 0.9) per 1 point increase, *p* < 0.001) and having ever used alcohol (AOR 3.8 (1.8, 7.8), *p* < 0.001). Self-esteem z-scores for both PHIV and HIV negative participants were 1.9 standard deviations below the mean for population norms.

**Conclusions:**

Self-harm is common among PHIV and HIV negative adolescents in England. Reassuringly however, they do not appear to be at an increased risk compared to the general adolescent population (15–19% lifetime prevalence). The low level of self-esteem (compared to available normative data) in both groups is worrying and warrants further attention.

## Introduction

Advancements in the medical care of children with perinatally acquired human immunodeficiency virus (HIV) have substantially improved their prognosis. As a result, large numbers of children living with HIV are now maturing into adolescence and early adulthood [1]. Adolescence is a time when many mental health problems, as well as behaviours that may increase the risk of mental health problems, emerge [2]. Children growing up with a life threatening and often highly stigmatised infection such as HIV may be at a particularly high risk for mental health problems, including self-harm and suicide.

Self-harm refers to an intentional act of self-injury or self-poisoning carried out by an individual, irrespective of motivation [3]. It includes behaviours such as cutting, overdose or self-poisoning, self-battering and burning [4]. In large population-based studies, of those adolescents reporting self-harm, cutting was identified as the most prevalent method and repeat episodes of self-harm were common [5–7]. Furthermore, very few of those who self-harm present to hospital for medical attention [8]. Whilst it is clear that not all self-harm is suicidal in nature, a follow-up study of over 40,000 patients presenting to hospitals in England found the age-adjusted risk of completed suicide in the first year following self-harm to be 49 times higher than in the general population [9]. Self-harm was identified as an emerging problem in the United Kingdom (UK) in the 1960s [10]. There has however, been renewed prioritisation on understanding suicidal phenomena such as self-harm as suicide among adolescents has become increasingly prominent, now the second most common cause of mortality in 15–29 year olds [11] and the most common cause of death in females aged 15–19 globally [4].

Numerous risk factors and correlates of self-harm have been identified. The most vulnerable age for initiating self-harm behaviours seems to be between 11 and 14 years [6, 12, 13], with females more at risk than males [4]. Whilst ethnicity has not consistently been identified as a risk factor for self-harm [14], there is some evidence that young black females are a particularly high risk group in the UK [15]. Furthermore, underlying psychological variables such as impulsivity, low self-esteem, depression, and anxiety have all been associated with self-harm [16]. Finally, a number of studies have found an association between suicidal phenomena in adolescents and alcohol and drug use [17].

In the last 5 years, three large studies have estimated prevalence of self-harm in adolescents in England using anonymous self-completed questionnaires. In a study of almost 4000 12–16 year olds recruited from schools, Stallard et al reported a 15% prevalence of self-harm in the preceding year [6]. In a further study of 2000 adolescents aged 13–18 years recruited by a market research agency from across England a lifetime prevalence of 15.5% was reported [12]. Finally, the Avon Longitudinal Study of Parents and Children (ALSPAC) birth cohort reported an 18.8% lifetime prevalence of self-harm among almost 5000 16–17 year old participants [5]. A recent UK study utilising national databases to investigate trends over time reported an increase in the incidence of self-harm from 45.9 per 10,000 in 2011 to 77.0 per 10,000 in 2014 among girls aged 13–16 [18].

Similar rates of self-harm have been reported across Europe. In the Child and Adolescent Self-harm in Europe (CASE) study, among more than 30,000 15–16 year olds from 7 different European countries, a lifetime history of self-harm was reported by 13.5 and 4.3% of females and males respectively [7].

Very few studies have reported prevalence of self-harm and suicide among young people with perinatally-acquired HIV (PHIV). One study of psychiatric hospitalisation among PHIV children < 15 years of age in the USA found that 18.7% of participants who were hospitalised during the study period were admitted because of suicidal attempts or ideation [19]. Further evidence from Kenya identified suicidality but no previous suicide attempts in 18% of the 162 6–18 year old participants with PHIV [20]. In a case-control study of 10–17 year old children from Rwanda, over 20% of the 218 HIV positive and 228 HIV negative children with a caregiver living with HIV had engaged in suicidal behaviour in the preceding 6 months [21]. In this study the odds of suicidal behaviour almost doubled among those living with HIV compared to the 237 HIV negative children with no HIV in their family (OR 1.85, 95% confidence interval (CI): 1.12, 3.05, 21). Finally, in the first study to report on mortality outcomes post-transition to adult care for young people with PHIV in the UK, suicide was the cause of two out of 11 deaths [22]. On the basis of this limited evidence it seems likely that suicidal ideation and self-harm are common amongst adolescents with PHIV, and since self-harm has been repeatedly identified as the strongest risk factor for subsequent suicide [23] there is an urgency to investigate the prevalence of, and the associated risk factors for self-harm in this population.

A number of studies investigating the effect of HIV infection on adolescent mental health lack an appropriate comparator group. However, mental health outcomes are affected by a complex interaction of genetic, biomedical, psychosocial and environmental influences [24]. For example, PHIV children have at least one biological parent living with HIV, and are therefore more likely to experience parental illness or even death, which may result in multiple caretaking transitions [25]. In the UK, a high proportion of PHIV children were born abroad (mainly in sub-Saharan Africa), and may therefore be exposed to the stressors of immigration and cultural adjustment [1, 26]. Therefore a comparator group which can approximate the life experiences of growing up with HIV in the household would help to isolate the effect of HIV infection itself on mental health outcomes of young people with PHIV.

The objective of this study was to determine the prevalence of self-harm among young people with PHIV and an appropriate comparison group of HIV negative young people living in England.

## Methods

The Adolescents and Adults Living with Perinatal HIV (AALPHI) cohort study is evaluating the impact of HIV infection and ART exposure on young people with PHIV and HIV negative young people in England. Participants were enrolled from HIV clinics and community services in England between 2013 and 2015. Detailed methods have been described previously [27]. All participants with PHIV had known their HIV status for at least 6 months and were aged 12–21 years. HIV negative young people were aged 13–23 years, had a negative point-of-care HIV test result at interview, and had either a sibling or parent with HIV. All participants had lived in the UK for 6 months or longer, could speak and understand English, and were able to give informed consent to participate in the study.

Participants underwent a two-hour face-to-face interview with a trained research nurse. Computer-Assisted Self-Interview (CASI) was used for sensitive questions. A series of questions assessed self-harm (the first question being “Have you ever hurt yourself on purpose in any way (e.g. by taking an overdose of pills, or by cutting yourself)?”), including the frequency and type of self-harm behaviours, as well as the presence or absence of suicidal intent during self-harming. Questions were adapted from the Avon Longitudinal Study of Parents and Children (ALSPAC) [5, 28, 29].

Levels of anxiety and depression symptoms were measured using the Hospital Anxiety and Depression Scale (HADS) [30], and standard definitions were used for categories. Self-esteem was measured using the Rosenberg self-esteem scale (scores range from 0 to 30: higher scores indicating better self-esteem) [31]. Health-related quality of life (HRQL) was measured using the Pediatric Quality of Life Inventory (PedsQL™ 4.0) (Teenage report for 13–18 and Young Adult report for 18–25 years), and a composite total score ranged between 0 and 100 (with higher scores representing better HRQL). Body image satisfaction was measured using four questions from the Minneapolis-Manchester Quality of Life Instrument (MMQL) [32] body image domain. Scores range from 0 to 100 (with higher scores indicating greater satisfaction with physical appearance.) For each test z-scores were calculated from normative data [33–36], with scores above zero indicating better clinical status. Major life events were assessed using a 21 item checklist, used in the ALSPAC study, which included events related to family, friends, and self. Higher total scores indicate less distress from significant life events in the preceding year. Normative data were not available for this measure and so z-scores were not calculated.

Data were analysed using STATA version 14 (Stata Corp, College Station, Texas, USA). Chi squared and Fisher’s exact tests were used to compare proportions; Wilcoxon rank sum and t-tests compared median and means respectively. Results are presented for non-missing values; missing values were less than 10% of study participants unless specified. The effect of potential predictors of self-harm were explored using logistic regression modelling. Factors considered a priori to be associated with self-harm for all participants were age, sex and HIV status. Other variables considered were: sociodemographic (ethnicity, birth outside the United Kingdom/Ireland); social/ family factors (currently living with parents/carers, living in original nuclear family (both biological parents), parent or caregiver employed, death of one or both parents, number of main carers (adults taking responsibility for the participant during childhood), previous contact with social services or educational support services); mental health factors (HRQL, Rosenberg self-esteem, body image satisfaction score, HADS depression and anxiety, and effects of major life events scores); and lifestyle/behavioural factors (ever alcohol, ever cannabis, ever recreational drug use other than cannabis). Variables with *p* < 0.15 in univariable analysis were considered in multivariable analysis using backward selection, and a two-tailed *p* < 0.05 was considered statistically significant.

An additional model for only the PHIV young people considered the additional effect of CD4 count and viral load suppression < 50 c/ml within 6 months of interview, self report of antiretroviral therapy adherence (not missing any doses in the last 3 days or more than two consecutive days in the last month), age at HIV disclosure, and current feelings about HIV diagnosis (5-item visual analogue scale to score how upset, sad and alone participants felt in relation to their HIV diagnosis, as well as how much they think about their diagnosis and worry about their future health), on levels of self-harm. For this analysis HIV-related variables were obtained through linkage to the Collaborative HIV Paediatric Study (CHIPS) database [26].

Full ethical approval for the AALPHI study was obtained from Leicester Research Ethics Committee.

## Results

### Sample characteristics

A total of 303 PHIV and 100 HIV negative participants had self-harm data. Of the HIV negative participants, 47.0% had PHIV siblings (39 of whom were in AALPHI), 49.0% had a mother living with HIV, and 4.0% had a close friend with PHIV. Sociodemographics of the two groups were similar (Table 1). A slightly higher proportion of PHIV compared to HIV negative participants were male (40.9% vs. 31.0%, *p* = 0.077) but their age distribution was similar, with a median age overall of 16.7 years. A higher proportion of PHIV participants were black African (85.8% vs. 73.0%, *p* = 0.003) and were born outside the UK (58.4% vs. 47.0%, *p* = 0.046). The majority of participants were living at home with parents or caregivers (90.3% overall), but far fewer were living in their original nuclear family (21.1% overall).

**Table 1:** Participant characteristics by HIV status

	Total (*n* = 403)	PHIV (*n* = 303)	HIV- (*n* = 100)	*P* value
	n (%) or mean {SD} or median [IQR]			
Sociodemographics				
Male sex	155 (38.5)	124 (40.9)	31 (31.0)	0.077
Age group				
≤ 15 years	157 (39.0)	115 (38.0)	42 (42.0)	0.541
16–18 years	160 (39.7)	125 (41.2)	35 (35.0)	
≥ 19 years	86 (21.3)	63 (20.8)	23 (23.0)	
Age, median [IQR]	16.7 [15.0, 18.6]	16.7 [15.1,18.5]	16.7 [14.5,18.7]	0.770
Black ethnicity	333 (82.6)	260 (85.8)	73 (73.0)	0.003
Born outside UK/Ireland	224 (55.6)	177 (58.4)	47 (47.0)	0.046
Social/ family factors				
Live with parents/carers	363 (90.3)	274 (90.7)	89 (89.0)	0.613
Live in original nuclear family	85 (21.1)	69 (22.9)	16 (16.0)	0.146
Parent/carer employed	274 (69.9)	217 (73.6)	57 (58.8)	0.006
Biological parent vital status:				
Both parents alive	249 (65.7)	178 (62.7)	71 (74.7)	0.093
One parent deceased	112 (29.6)	92 (32.4)	20 (21.1)	
Both parents deceased	18 (4.8)	14 (4.9)	4 (4.2)	
Number of carers^a^	1 [1.0,2.0]	1 [1.0,2.0]	1 [1.0,2.0]	0.297
Ever contact with social services	112 (28.4)	83 (27.8)	29 (30.2)	0.643
Ever contact with educational support services	103 (25.8)	84 (28.0)	19 (19.0)	0.075
Mental health factors				
PedsQL HRQL score	76.7 {14.0}	75.6 {13.9}	79.8 {13.7}	0.009
z score	−0.7 {1.2}	−0.8 {1.2}	−0.4 {1.2}	0.007
Rosenberg self-esteem score	20.6 {5.3}	20.6 {5.2}	20.7 {5.5}	0.977
z score	−1.9 {1.5}	−1.9 {1.5}	−1.9 {1.6}	0.953
MMQL body image satisfaction score	64.3 {23.2}	64.4 {22.6}	63.9 {25.2}	0.850
z score	−0.2 {0.9}	−0.2 {0.8}	−0.2 {0.9}	0.850
HADS Depression score	3.8 {3.1}	3.9 {3.1}	3.5 {2.9}	0.245
z score	−0.1 {0.8}	−0.1 {0.8}	0.0 {0.8}	0.154
HADS Anxiety score	6.4 {4.0}	6.5 {3.9}	6 {4.1}	0.268
z score	−0.1 {0.9}	0.0 {0.8}	−0.2 {0.9}	0.133
Major life events	−0.1 {2.7}	0.0 {2.6}	−0.4 {3.0}	0.270
Lifestyle/ behavioural factors				
Ever used alcohol	163 (42.5)	120 (41.5)	43 (45.3)	0.522
Ever used cannabis	68 (18.2)	42 (14.8)	26 (28.6)	0.003
Ever used other recreational drugs	15 (4.0)	10 (3.5)	5 (5.5)	0.373

*PHIV* perinatally-acquired HIV, *HIV-* HIV negative

^a^number of adults taking responsibility for the participant during childhood

Mental health parameters were generally comparable between the two groups, with the exception of HRQL scores, where PHIV participants’ z-scores were lower than HIV negative participants (− 0.8 vs. -0.4, *p* = 0.007). Z-scores for anxiety, depression and body image satisfaction for both groups were close to zero, indicating similarity to normative data, however participants from both groups had significantly lower self-esteem z-scores compared with normative data (− 1.9 (standard deviation (SD) 1.5) overall).

### Prevalence of self-harm

A lifetime history of self-harm was reported in 13.9% (56/403) of participants overall, and 12.2% of PHIV participants and 19.0% of HIV negative participants (*p* = 0.089). Of those who had self-harmed, almost half (25/53, 47.2%) had done so in the 12 months preceding the interview. Of these participants 36.0% (9/25) reported only one episode of self-harm in the last year, and the remainder reported 2–5 episodes (11/25, 44.0%) or > 5 episodes (5/25, 20.0%). Forty-four per cent (11/25) of those who self-harmed in the last 12 months were aged ≤15 years, 36% (9/25) 16–18 years, and 20% (5/25) ≥19 years, and 92% (23/25) were female. The most common methods of self-harm overall were cutting, either alone or in combination with other methods (27/56, 48.2% and 8/56, 14.3% respectively), and swallowing pills or other poisonous substances (15/56, 26.8% alone and 5/56, 8.9% in combination). After the last episode of self-harm, the majority (43/56, 76.8%) did not require any medical attention but 14.3% (8/56) sought medical assistance at hospital emergency departments (Table 2).

**Table 2:** Prevalence of self-harm thoughts and actions by presence of suicidal intent

Variable	Total (*n* = 56)	Self-harm without suicidal intent (n = 30)	Self harm with suicidal intent (*n* = 26)	*P* value
	n (%)			
Thoughts				
Ever felt life was not worth living?	47 (83.9)	21 (70.0)	26 (100.0)	0.002
Ever wished you were dead and away from it all?^a^	41 (87.2)	16 (76.2)	25 (96.2)	0.041
Actions				
Repeated self-harm^b^	16 (64.0)	4 (44.4)	12 (75.0)	0.127
Swallowed pills/ poisonous substance at last self-harm	20 (35.7)	9 (30.0)	11 (42.3)	0.338
Cutting at last self-harm	35 (62.5)	20 (66.7)	15 (57.7)	0.489
Presented to hospital after last self-harm	8 (14.3)	2 (6.7)	6 (23.1)	0.166

^a^unknown for 9 self-harm without suicidal intent; ^b^ > 1 episode, of those who had self-harmed in the preceding 12 months (*N* = 25 overall, *N* = 9 and 16 without or with suicidal intent respectively)

### Prevalence of suicidal intent in those who had self-harmed

Just under half (26/56, 46.4%) of those who had self-harmed reported suicidal intent at any previous episode of self-harm, and nearly all of these participants reporting feeling their life was not worth living and they wished they were dead and away from it all (Table 2). There were no differences in repeated self-harm, method of self-harming, number who went to hospital (Table 2), sociodemographic or social/family factors (data not shown) between those who had self-harmed with and without suicidal intent. However, a higher proportion of those who reported suicidal intent had prior contact with social services (15/26, 57.7%) compared to those without suicidal intent (6/29, 20.7% respectively, *p* = 0.05).

### Self-harm and mental health scores

**Fig. 1:**
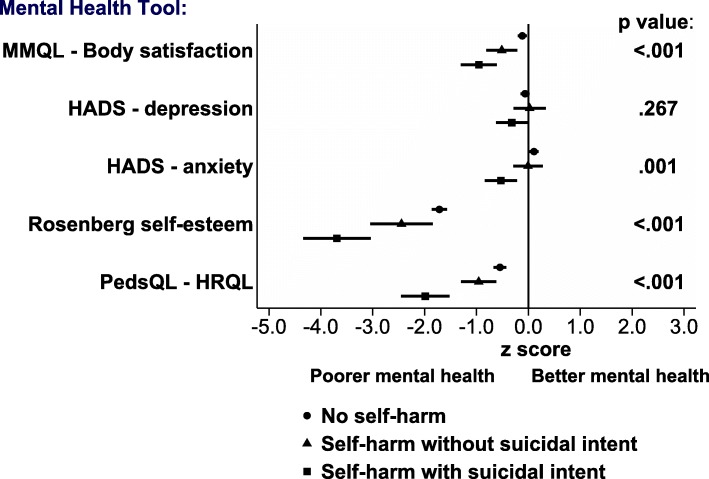
Mental health test z-scores and self-harm with and without suicidal intent

Figure 1 presents mean z-scores for each mental health test for participants with no history of self-harm, self-harm without suicidal intent, and self-harm with suicidal intent. Mean z-scores for all three groups were below reference means for body image satisfaction score, self-esteem and quality of life. Anxiety and depression scores for the three groups were closer to zero. With the exception of depression score, there was a consistent trend for a progressive reduction in z-score across the groups, with those reporting no self-harm having the highest z-scores, and those reporting self-harm with suicidal intent the lowest z-scores. Mean (SD) raw scores for the major life events variable were consistent with this trend (no self-harm 0.1 (2.7), self-harm with no suicidal intent − 0.3 (2.9), self-harm with suicidal intent − 2.1 (2.5).

### Predictors of self-harm

Table 3 presents univariable and multivariable predictors of self-harm. Before and after adjustment for other variables, HIV status was not associated with self-harm (adjusted *p* = 0.212). Prevalence of self-harm was higher in females (20.6%) compared to males (3.2%) and after adjustment, the odds of self-harm were 5 times greater in females than males (adjusted odds ratio (AOR) 5.3, 95% CI 1.9, 14.1, *p* = 0.001). Each 1-point increase in the Rosenberg self-esteem score was associated with a 10% reduction in adjusted odds of self-harm (95% CI: 0.8, 0.9, *p* < 0.001). The prevalence and odds of self-harm were also higher in those who had ever used alcohol (AOR 3.8, 95% CI: 1.8, 7.8, p < 0.001). No other factors were associated with self-harm in multivariable analysis. Two sensitivity analyses, one including only female participants, and another including Rosenberg self-esteem z-scores, found no difference in predictors of self-harm (data not shown). In the separate model for PHIV young people, multivariable predictors of self-harm were the same as the overall model, with similar effect size (data not shown).

**Table 3:** Prevalence and predictors of self-harm

Variable		Prevalence	Univariable		Multivariable	
		n/total (%)	OR (95%CI)	*P* value	OR (95%CI)	*P* value
A priori factors						
HIV status	Positive (PHIV)	37/303 (12.2)	1		1	
	Negative (HIV-)	19/100 (19.0)	1.7 (0.9, 3.1)	0.091	1.6 (0.8, 3.1)	0.212
Sex	Male	5/155 (3.2)	1		1	
	Female	51/248 (20.6)	7.8 (3.0, 19.9)	< 0.001	5.3 (1.9, 14.1)	0.001
Age	≤15 years	15/157 (9.6)				
	16–18 years	27/160 (16.9)				
	≥19 years	14/86 (16.3)				
	Per 1 year inc.		1.1 (1.0, 1.2)	0.214	1.0 (0.9, 1.2)	0.924
Other factors						
Social services support	No	34/283 (12.0)	1			
	Yes	21/112 (18.8)	1.7 (0.9, 3.1)	0.084		
PedsQL (HRQL score)	≥median	14/210 (6.7)				
	<median	42/191 (22.0)				
	Per 25 point inc.		0.3 (0.2, 0.5)	< 0.001		
Rosenberg self-esteem	≥median	14/240 (5.8)				
	<median	42/162 (25.9)				
	Per 1 point inc.		0.9 (0.8, 0.9)	< 0.001	0.9 (0.8, 0.9)	< 0.001
MMQL Body image	≥median	19/232 (8.2)				
	<median	36/152 (23.7)				
	Per 25 point inc.		0.5 (0.3, 0.6)	< 0.001		
HADS Anxiety score	Normal (< 8)	22/234 (9.4)				
	Mild (8–10)	19/80 (23.8)				
	Moderate (11–14)	9/50 (18.0)				
	Severe (≥15)	3/12 (25.0)				
	Per 1 point inc.		1.1 (1.1, 1.2)	0.001		
Major life events	≥median	27/250 (10.8)				
	<median	29/153 (19.0)				
	Per 1 point inc.		0.9 (0.8, 0.9)	0.002		
Ever used alcohol	No	17/221 (7.7)	1			
	Yes	37/163 (22.7)	3.5 (1.9, 6.5)	< 0.001	3.8 (1.8, 7.8)	< 0.001
Ever cannabis	No	35/306 (11.4)	1			
	Yes	17/68 (25.0)	2.6 (1.3, 5)	0.004		
Ever other drugs	No	48/359 (13.4)	1			
	Yes	4/15 (26.7)	2.4 (0.7, 7.7)	0.156		

Only factors associated with self-harm at univariable (*p* < 0.15) or multivariable (*p* < 0.05) level are shown

*PHIV* perinatally-acquired HIV, *HIV-* HIV negative

## Discussion

The overall results from this study are commensurate with results from a number of large population studies on self-harm in adolescents both in the UK and internationally. This study found an overall prevalence of self-harm of 13.9%, with girls carrying a disproportionate burden. One in five girls in the AALPHI study reported at least one episode of self-harm, and nearly all (23/25) of those who self-harmed in the preceding 12 months were female. Furthermore, this study found similar patterns of self-harm behaviour to those described in the existing literature, with a predominance of self-cutting (62.5%), high levels of repetition (64.0%), and low levels of help seeking behaviour at hospitals or emergency department after self-harm (14.3%). This reaffirms the evidence that self-harm is a common problem in adolescent community populations in the UK and is mostly ‘hidden’ from health professionals, although whether young people with PHIV in our study had discussed self-harm with staff at their HIV clinic is unknown.

Importantly, our study did not identify significant differences in prevalence of self-harm in the AALPHI cohort between those participants living with perinatal HIV and those who were HIV negative, suggesting that the impact of HIV infection itself on mental health outcomes may be less important than the psychosocial milieu in which these children are raised. The only other study to date to specifically address suicidal ideation and behaviour in young people living with HIV also found no difference in prevalence of suicidal behaviour between HIV positive and HIV negative affected (seronegative with an HIV positive care-giver) adolescents living in Rwanda (21.5 and 21.1% respectively) [21]. It did however find significantly higher levels of suicidal behaviour among adolescents living with HIV and children affected by HIV in comparison to HIV negative children with no HIV in their family (OR 1.9, 95% CI 1.1, 3.1, and OR 1.9, 95% CI 1.2, 3.1 respectively). In contrast, our study’s prevalence of self-harm was similar to that reported in the general UK adolescent population in two large community based studies [5, 12].

Our study found a strong association between self-esteem and self-harm. Each one point increase in Rosenberg score was associated with a 10% reduction in odds of self-harm (95% CI: 0.8, 0.9, *p* < 0.001). Similarly the CASE study found a significant difference in self-esteem scores between those with no history of self-harm and those with single and multiple episodes of self-harm, in the general adolescent population across Europe [16]. Furthermore, a recent study of nearly 9000 13–19 year olds from Norway found higher self-esteem was strongly associated with reduced risk of self-harm hospitalisation [37]. Importantly, self-esteem z-scores for both groups in AALPHI were almost 2 standard deviations below the mean for general adolescent populations, possibly highlighting a particular mental health vulnerability in this population.

Our study did not find an association between self-harm and depression. This is in contrast to much of the existing literature. The Rwandan study reported an AOR of 1.8 (95% CI 1.1, 3.0, *p* = 0.003) for suicidal behaviour in adolescents with self-reported scores above the diagnostic cut-off for depression compared to those below the cut-off score [21]. The ALSPAC study found a greater than 5 times higher odds of self-harm among participants who scored above diagnostic cut-off for depressive symptoms than those who scored below [5]. Similarly the CASE study described a dose-response relationship whereby higher scores of depression were associated with worsening history of self-harm – from no self-harm, to self-harm thoughts only, to a single and then multiple episodes of self-harm [7]. The lack of association between depression and self-harm in our study was thus unexpected. It should not simply be dismissed as measurement artefact, as although some have criticised the HADS tool [38], the CASE study did find an association between depression and self-harm using the HADS [16].

Another possibility is that our study did not detect an effect of depression due to its relationship with self-esteem, which itself was a strong predictor of self-harm. Low self-esteem has been found to both increase an individual’s vulnerability to depression, and to be a symptom of depression [17], and we recently found that lower self-esteem predicted higher depression scores in the AALPHI cohort [39]. Previous research has shown that low self-esteem and depressive symptoms often co-occur among the general population of adolescents [40, 41], although some studies suggest that the direction of the association between self-esteem and depressive symptoms is predominantly from self-esteem to depressive symptoms [42, 43]. Therefore it might be the case that some of the adolescents in our study with low self-esteem are yet to develop depressive symptoms.

A number of studies that found strong association between self-harm and depression, including ALSPAC and the Rwandan study, did not measure self-esteem [5, 21]. However, the CASE study, which examined both self-esteem and depression, found an independent association with self-harm for both [16]. In our study depression was not significantly related to self-harm in the univariable model, or in a sensitivity analysis replacing self-esteem with depression in the multivariable model. A possible reason for this is that the prevalence of depression overall was relatively low in our study (84% had a normal HADS depression score) compared to other studies, and therefore an association with self-harm may be harder to detect. The CASE study which also measured depression using HADS reported an overall mean (SD) depression score of 4.4 (3.3, 16) compared to 3.8 (3.1) in our study. Furthermore whilst different measures of depression were used in other studies, the prevalence of depression overall was 26% in the Rwandan study [21], and 45% the in self-harm group in ALSPAC [5] compared to 23% in AALPHI.

A further possible explanation for the lack of association between depression and self-harm in our study is that through the clinical management of HIV and the required contact with health professionals there may be some mitigation of mental health vulnerabilities [44]. To date, research into factors that protect against self-harm is extremely limited [45], so it is uncertain whether contact with health professionals does indeed have any affect. However, if this were the case one would expect higher prevalence of self-harm or depression in the HIV negative comparison group. The AALPHI comparator group did in fact have higher prevalence of self-harm (19.0% vs. 12.2%) however the difference was not statistically significant (*p* = 0.089), and there was no difference in the level of depression between the two groups in a previous analysis [39].

Finally, our study also found a step-wise deterioration in all mental health parameters (with the exception of depression) as we compared the non-self-harm group to the self-harm without suicidal intent, and the self-harm with suicidal intent group. Whilst there is controversy over the clinical usefulness of differentiating self-harm with suicidal intent [46], this finding is interesting. It suggests that rather than representing separate entities such as non-suicidal self-injury and attempted suicide, that self-harm with and without suicidal intent exist on a spectrum and may be affected by similar underlying mental-health parameters.

This study is important as it is the first in the UK to address the issue of self-harm among young people living with HIV. The inclusion of an appropriate comparator group enables the distinction between effects of HIV infection itself from the surrounding psychosocial environmental influences. Using definitions of self-harm consistent with both the ALSPAC and CASE studies allowed for comparison with these larger general population samples. Even though the epidemiology of HIV varies markedly around the world, and many environmental factors impact on mental health, this study showed that young people growing up with HIV in England are at similar risk of self-harm to young people growing up with HIV in other countries and to the adolescent population generally.

The study also has a number of limitations. Firstly, the reliance on self-report assessment tests introduces the possibility that participants may give false or inaccurate responses, or choose not to respond at all. However, we aimed to minimise this through ensuring anonymity of questionnaires as well as the use of CASI for sensitive questions, and the number of missing responses was very low. Secondly as AALPHI is a larger study addressing multiple domains, we were constrained by interview length, and thus used a sub-set of the self-harm questions from the ALSPAC study. As a result we could not investigate the reasons for self-harm, nor were we able to identify those who had self-harm thoughts but no actions. Thirdly, there were a number of challenges to selecting mental health measurement tools that could be compared to existing literature. Not only is there great variability with regards to measures of mental health parameters used in existing literature, but not all tools have been validated for adolescent populations or have appropriate normative data available. Lastly, although all PHIV young people were invited to join AALPHI, some who self-harm may not have consented to take part. However we have previously reported that those who participated were broadly representative of young people in the national UK and Ireland paediatric cohort [26].

Despite being seemingly at high risk for self-harm, the comparable prevalence of self-harm in PHIV young people to HIV negative adolescents suggest that there could be protective factors moderating risk. Identification of these factors could assist with planning community suicide and self-harm prevention programmes. There is a poor evidence base for the effectiveness of interventions to improve self-esteem in HIV positive adolescents. One randomised trial in rural Zimbabwe found that engagement with community adolescent treatment supporters improved linkage to services, retention in care, ART adherence and psychosocial wellbeing [47]. Parental support within the family may also be a contributing factor in developing self-esteem in adolescents in the general population [48], and may be pertinent for young people affected by HIV in our study, some of whose parents had died. Evidence from other disease areas suggests that psychosocial interventions such as cognitive behavioural therapy and family therapy improve psychological outcomes [49]. In terms of day to day care of PHIV in the UK, the Children’s HIV Association (CHIVA) has Standards of Care which every child living with HIV should expect to receive, and these highlight the importance of psychology support as an integrated service [50]. Additionally CHIVA has a Family Conference each year, for parents, carers and family members of children and young people growing up with HIV, with a focus on achieving health and wellbeing, in addition to its annual residential support camp for children aged 11–16 years [51, 52].

As the prevention of mother-to-child transmission programmes improve and expand worldwide, our findings reiterate the importance of increasing awareness and addressing the mental health vulnerabilities of the growing population of HIV negative young people who have been exposed to HIV, who are not routinely in medical care. A greater understanding of the factors that both promote and prevent self-harm in this population could assist with the elucidation of the underlying aetiology of this worrying behaviour in the general adolescent population. Specifically, the identification of poor self-esteem among these adolescents, compared to the general adolescent population, warrants further investigation. In conclusion, this study of PHIV adolescents and a comparable group of HIV negative young people in the UK found levels of self-harm similar to the general adolescent population, and particularly high among female participants. Self-harm was also found to be significantly associated with worse self-esteem and having ever drunk alcohol.

## Data Availability

The AALPHI data are held at MRCCTU at UCL, which encourages optimal use of data by employing a controlled access approach to data sharing, incorporating a transparent and robust system to review requests and provide secure data access consistent with the relevant ethics committee approvals. The rationale for this approach has been published (doi: 10.1186/s13063-015-0604-6). Ethics committee approval for use of AALPHI data is restricted to specific approved protocols. All requests for data are considered and can be initiated by contacting mrcctu.ctuenquiries@ucl.ac.uk.

## Abbreviations

AALPHI: Adolescents and Adults Living with Perinatal HIV
ALSPAC: Avon Longitudinal Study of Parents and Children
AOR: adjusted odds ratio
CASE: Child and Adolescent Self-harm in Europe
CASI: Computer-Assisted Self-Interview
CHIPS: Collaborative HIV Paediatric Study
CI: Confidence interval
HADS: Hospital Anxiety and Depression Scale
HIV: Human immunodeficiency virus
HRQL: Health-related quality of life
IQR: Interquartile range
MMQL: Minneapolis-Manchester Quality of Life Instrument
OR: Odds ratio
PedsQL: Pediatric Quality of Life Inventory
PHIV: Perinatally-acquired HIV
SD: Standard deviation
UK: United Kingdom
